# ORAI1 CRAC Channel in Immune Cell is a Therapeutic Target for Pancreatitis-Associated Acute Lung Injury

**DOI:** 10.1093/function/zqad065

**Published:** 2023-11-24

**Authors:** Shuang Peng, Lu Ke, Weiqin Li

**Affiliations:** School of Sport and Health Sciences, Guangzhou Sport University, Guangzhou 510500, China; Key Laboratory of Sports Technique, Tactics and Physical Function of General Administration of Sport of China, Scientific Research Center, Guangzhou Sport University, Guangzhou 510500, China; Department of Critical Care Medicine, Jinling Hospital, Affiliated Hospital of Medical School, Nanjing University, Nanjing 210002, Jiangsu, China; National Institute of Healthcare Data Science, Nanjing University, Nanjing 210010, Jiangsu, China; Department of Critical Care Medicine, Jinling Hospital, Affiliated Hospital of Medical School, Nanjing University, Nanjing 210002, Jiangsu, China; National Institute of Healthcare Data Science, Nanjing University, Nanjing 210010, Jiangsu, China

**Keywords:** Ca^2+^ signaling, ORAI1, neutrophils, acute lung injury, acute pancreatitis

## A Perspective on “Neutrophil-Specific ORAI1 Calcium Channel Inhibition Reduces Pancreatitis-Associated Acute Lung Injury”

Acute pancreatitis (AP) is initiated within pancreatic exocrine cells and sustained by dysregulated systemic inflammatory responses.^1^ Mounting evidence confirms that abnormal intracellular Ca^2+^ signals causing Ca^2+^ overload and injury in pancreatic acinar cells initiate AP.^2^ Excessive store-operated Ca^2+^ entry through Ca^2+^ release activated Ca^2+^ (CRAC) channels of the ORAI1 type in pancreatic acinar, stellate, and immune cells is a key trigger of AP.^2^, ^3^ Previous studies have shown that ORAI1 inhibitors prevented and/or markedly ameliorated AP in mice, primarily through the prevention of Ca^2+^-dependent pancreatic acinar cell death.^2–5^ Because of these findings, ORAI1 inhibitors are currently under active clinical development to treat AP.^6^ Although the acinar cells have traditionally been the primary focus when considering the pathophysiology of AP, it has been proposed that the primary Ca^2+^-dependent injury of the acinar cells triggers processes in adjacent stellate and immune cells that create necrotic amplification loops.^2^ Nevertheless, little is still known about ORAI1 cell-specific effects.

Macrophages and neutrophils are major immune cell types infiltrating the pancreatic tissue in the early stage of AP.^7–9^ With regard to macrophage function, it has been proposed that Ca^2+^ signals in these cells, elicited by action on purinergic receptors due to the release of adenosine triphosphate/adenosine diphosphate (ADP/ATP) from dying acinar cells, will amplify necrotic processes in the acinar cells, thereby worsening AP.^9^

**Figure 1.**
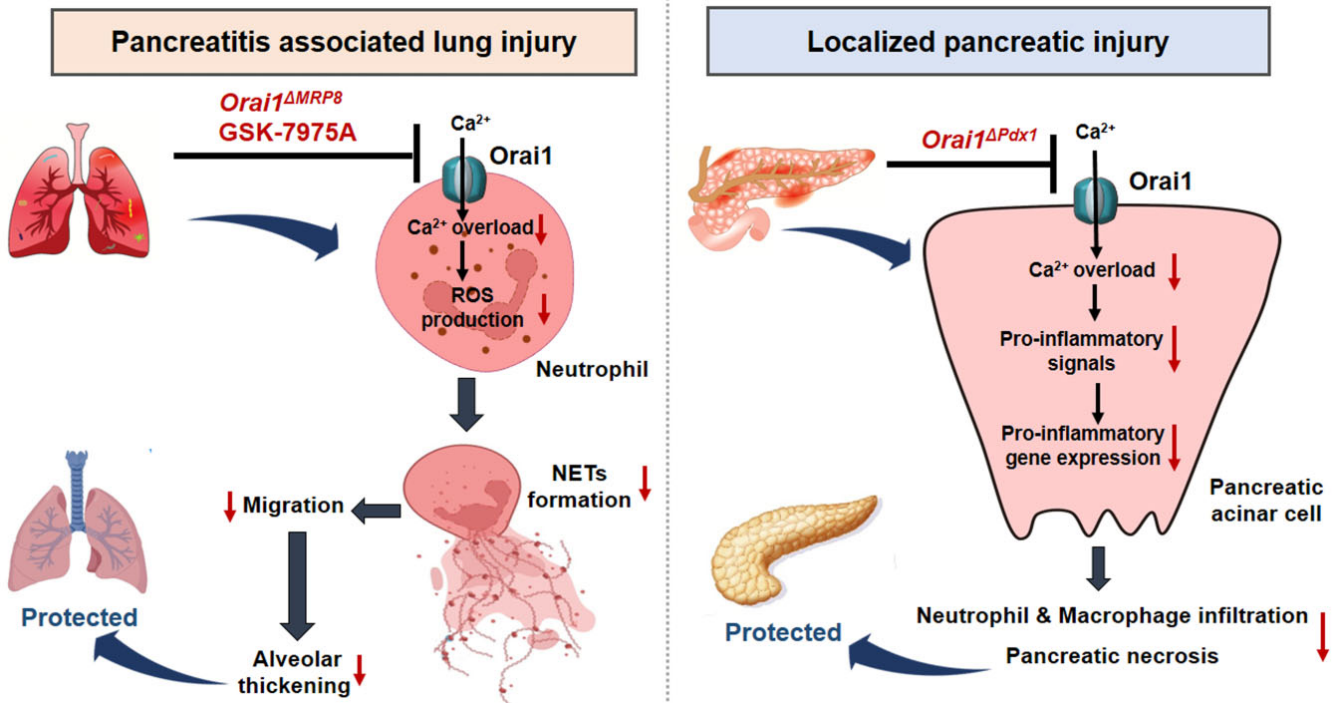
Mechanisms of the cell-specific effects of ORAI1 on the development of acute pancreatitis and pancreatitis-associated lung injury (see text for further explanation).

The most dangerous aspect of AP is the potential acute lung injury.^1–3^ However, the relationship between processes in the individual cell types in the pancreas and the lung injury have not yet been clarified. A new study published in this issue of *Function*^8^ focusses on the specific role of the neutrophils. Niu et al.^8^ present data that provide novel insights into the role of two different cell-specific ORAI1 channels (neutrophils versus pancreatic parenchymal cells) on pancreatitis-associated lung damage (Figure 1).

By crossing *Orai1^f/f^* mice with a pancreas-specific Cre line (Pdx1-Cre), pancreas-specific *Orai1* deficient (*Orai1^ΔPdx1^*) mice were created.^8^ Acute pancreatitis was induced by hyperstimulation of cholecystokinin (CCK) receptors on the acinar cells by the frog peptide caerulein (a CCK analogue) or retrograde biliopancreatic duct infusion of sodium taurocholate. The results demonstrated that the pancreatic injury is primarily dependent on pancreatic parenchymal ORAI1.^8^ Deletion of pancreatic parenchymal cell ORAI1 significantly reduced both macrophage and neutrophil infiltration in the pancreas but, importantly, not in the lung.^8^ This is in keeping with the primary role of the pancreatic epithelial cells in the initiation of AP and the major role of macrophages in pancreatic injury.^9^

By crossing *Orai1^f/f^* mice with a neutrophil-specific Cre line (MRP8-Cre), neutrophil-specific *Orai1* deficient mice were generated. Using genetic (*Orai1^ΔMRP8^*) or pharmacological inhibition (ORAI1 inhibitor, GSK-7975A), Niu et al. confirmed that ORAI1 in neutrophils is a crucial Ca^2+^ modulator mediating Ca^2+^ influx and multiple neutrophil functions, including migration, intracellular reactive oxygen species production, and neutrophil extracellular traps formation in both mouse and human neutrophils.^8^

Acute lung injury is the most common systemic complication of AP, and the neutrophil is the major infiltrating immune cell in the lung central to acute lung injury. Using various experimental models of AP, Niu et al. have demonstrated that deletion of neutrophil *Orai1* results in a significantly reduced neutrophil infiltration and alveolar membrane thickening in the lung, and although this did not affect the measure of lung permeability, lung cytokine expression was also reduced by reverse transcription-quantitative polymerase chain reaction (RT-qPCR) analysis of the lung tissue.^8^ Collectively, the new work^8^ demonstrates that different cell types contribute to local or systemic organ injury in AP, with parallel findings in human cells, further emphasizing that drugs that target common mechanisms in multiple rather than single cell types may be more effective in the treatment of AP.^2^, ^8^

The new results reported by Niu et al.^8^ indicate the importance of cell-specific intracellular networks that utilize the CRAC channel ORAI1. The CRAC channels in the pancreatic cells and those in the neutrophils would appear to have distinct and important roles in AP. It is, however, important to realize that although Niu et al.^8^ describe the results of what they label as deletions of ORAI1, these are not complete suppressions of the channel (only ∼70 reduction of expression). Furthermore, although ORAI1 may be the principal CRAC channel in neutrophils, there are others, notably ORAI2 and ORAI3, as well as transient receptor potential channels that may also allow Ca^2+^ entry. Clearly extensions of the work of Niu et al. to investigate the deletion of ORAI1 from other cell types, for example, in specific monocyte-macrophage lineages or pancreatic stellate cells,^10^ to inhibit Ca^2+^ entry may provide further important insights.

## Data Availability

There are no data presented in this perspective.
